# Pretreatment Attrition and Formal Withdrawal During Treatment and Their Predictors: An Exploratory Study of the Anxiety Online Data

**DOI:** 10.2196/jmir.2989

**Published:** 2014-06-17

**Authors:** Ali M AL-Asadi, Britt Klein, Denny Meyer

**Affiliations:** ^1^School of Health SciencesSwinburne University of TechnologyHawthornAustralia; ^2^Department of Arts and EducationGrande Prairie Regional CollegeGrande Prairie, ABCanada; ^3^DVC-Research & Innovation Portfolio and the School of Health Sciences & the Collaborative Research NetworkFederation UniversityBallaratAustralia; ^4^Centre for Mental Health ResearchThe Australian National UniversityCanberraAustralia; ^5^National eTherapy CentreSchool of Health SciencesSwinburne University of TechnologyHawthornAustralia

**Keywords:** pretreatment attrition, treatment withdrawal dropouts, predictors, anxiety disorders, eTherapy, e-mental health, Internet interventions

## Abstract

**Background:**

Although in its infancy, the field of e-mental health interventions has been gaining popularity and afforded considerable research attention. However, there are many gaps in the research. One such gap is in the area of attrition predictors at various stages of assessment and treatment delivery.

**Objective:**

This exploratory study applied univariate and multivariate analysis to a large dataset provided by the Anxiety Online (now called Mental Health Online) system to identify predictors of attrition in treatment commencers and in those who formally withdrew during treatment based on 24 pretreatment demographic and personal variables and one clinical measure.

**Methods:**

Participants were assessed using a complex online algorithm that resulted in primary and secondary diagnoses in accordance with the Diagnostic and Statistical Manual of Mental Disorders, Fourth Edition, Text Revision (DSM-IV-TR). Those who received a primary or secondary diagnosis of 1 of 5 anxiety disorders (generalized anxiety disorder, social anxiety disorder, obsessive-compulsive disorder, posttraumatic stress disorder, and panic disorder) were offered an online 12-week disorder-specific treatment program.

**Results:**

Of 9394 potential participants, a total of 3880 clients enrolled and 5514 did not enroll in one of the treatment programs following the completion of pretreatment assessment measures (pretreatment attrition rate: 58.70%). A total of 3199 individuals did not formally withdraw from the 12-week treatment cycle, whereas 142 individuals formally dropped out (formal withdrawal during treatment dropout rate of 4.25%). The treatment commencers differed significantly (*P*<.001-.03) from the noncommencers on several variables (reason for registering, mental health concerns, postsecondary education, where first heard about Anxiety Online, Kessler-6 score, stage of change, quality of life, relationship status, preferred method of learning, and smoking status). Those who formally withdrew during treatment differed significantly (*P*=.002-.03) from those who did not formally withdraw in that they were less likely to express concerns about anxiety, stress, and depression; to rate their quality of life as very poor, poor, or good; to report adequate level of social support; and to report readiness to make or were in the process of making changes.

**Conclusions:**

This exploratory study identified predictors of pretreatment attrition and formal withdrawal during treatment dropouts for the Anxiety Online program.

**Trial Registration:**

Australian and New Zealand Clinical Trials Registry ACTRN121611000704998; http://www.anzctr.org.au/trial_view.aspx?ID=336143 (Archived by WebCite at http://www.webcitation.org/618r3wvOG).

## Introduction

Although e-mental health interventions for the treatment of anxiety disorders have been shown to be effective [1-6] and increasing in popularity [7,8], research tends to be limited by high rates of attrition [8-10] and small to moderate sample sizes reported by most trials (see [11]). In addition, there appears to be little consensus regarding how attrition is defined in the literature. This lack of consensus is largely a result of each program offering different numbers of treatment modules and each study setting a different minimum number of treatment modules (or sessions) before a participant is considered a completer of a treatment program. Despite high rates of attrition, the majority of research published to date includes little or no analysis of relationships between demographic variables and attrition.

Attrition rates for e-mental health programs vary according to definition and treatment variables. Researchers report attrition rates for e-mental health treatment programs for anxiety or depression ranging from as high as 99% to as low as 1% [10-14] with the majority reporting attrition rates ranging between 20% and 40%. In a review of the effectiveness of e-mental health treatment programs for panic disorder, attrition rates for online therapy ranged between 4% and 36% [15]. Along similar lines, a recent examination of the efficacy of e-mental health programs for major depression, panic disorder, social phobia, and generalized anxiety disorder using a meta-analysis of 22 studies, revealed a range of 48% to 100% with a median of 80% of participants who began computerized cognitive behavioral therapy (CBT) completed all stages of their program [12].

Attrition appears to be greater in studies of Internet-based treatment that are open to the general public [16] and online therapy in which there is no therapist involvement [17,18] than in studies dealing with in-clinic samples and online samples in which assessors and therapists are involved. For instance, a study examining the usage and effectiveness of freely available, nontherapist-assisted, Internet-based CBT for panic disorder reported a very high attrition rate, with only 12 of 1161 registered users completing the 12-week therapy program [10]. In a study examining symptom change in people with anxiety and depression, the general public users completed significantly fewer symptom assessments than trial participants; out of 19,607 general public users, 12,141 (61.9%) completed at least 1 symptom assessment and 3055 (15.6%) completed 2 or more symptom assessments, whereas 157 of 182 (86.3%) trial participants completed at least 1 symptom assessment and 121 (66.5%) completed 2 or more symptom assessments [9]. Although demographic variables were collected, not many of these studies examined these variables in relation to attrition.

In clinical practice, attrition rates for the treatment of anxiety disorders vary by treatment setting and definition with a range from 10% to 60% [19-21]. Although females and older people are linked to greater participation [22], patients with milder anxiety disorder-specific symptoms, patients with higher levels of physical disability, women [23], and patients with a comorbid diagnosis [24], greater age and lower income [25], and lower level of education [26] have been linked to greater dropout rates. However, other researchers have reported no significant effect for all or some of these aforementioned variables [23,27-29]. Pretreatment attrition in clinic-based samples is typically higher than during treatment attrition [22,30], with more severe comorbid depressive symptoms and the presence of at least 1 or more children shown to be significant predictors of pretreatment attrition [23]. On the other hand, years of education, race, age, and employment status were significant predictors of attendance at the initial interview for treatment [31].

In summary, attrition rates have been reported for a large variety of treatment programs. It appears that attrition rates in both online and clinic-based samples are affected by treatment and demographic variables and tend to vary considerably depending on how attrition is defined. Although data on attrition rates for online and clinic-based treatment are widely available, research regarding the relationship between demographic variables and attrition for individuals participating in e-mental health treatment programs is inconclusive. Although more data are available regarding the demographic variables associated with attrition in clinic-based samples, the research that is available is inconclusive.

In this exploratory study, pretreatment attrition and formal withdrawal during treatment and their predictors are examined using data from the Anxiety Online platform. Because we have the number of participants who formally withdrew during treatment only and do not have data on how many modules each participant completed, this necessitated a different approach from the one outlined in the literature. To avoid any confusion, we elected to use the terms “formal withdrawal during treatment” rather than attrition and “no formal withdrawal during treatment” rather than completion.

**Figure 1:**
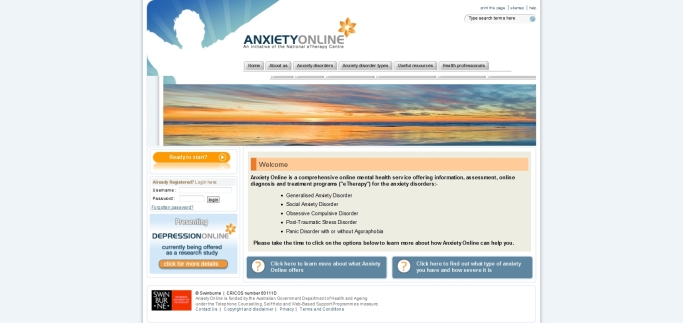
Homepage of Anxiety Online.

Anxiety Online is funded by the Australian Federal Government and is operated through the National eTherapy Centre at Swinburne University of Technology. This open access online service provides online assessment, including diagnosis of 21 mental health disorders in accordance with the *Diagnostic and Statistical Manual of Mental Disorders, Fourth Edition, Text Revision* (*DSM-IV-TR*), and self-guided and therapist-assisted treatment programs for the 5 anxiety disorders ([32]; Figure 1). It should be noted that the Anxiety Online platform was upgraded in September 2013 and now uses the name Mental Health Online [32].

This paper reports on one clinical measure and 24 demographic variables that are potentially associated with pretreatment attrition and formal withdrawal during treatment, in an effort to identify individuals who are likely to refuse treatment and individuals who formally withdraw from treatment prematurely. This is rather useful because identifying the characteristics that are more likely to lead to attrition and formally dropping out of treatment would make it possible to change aspects of the program to make it more engaging for these particular subgroups via tailoring and personalizing. In the absence of clear indicators as to the direction of associations between pretreatment attrition and formal withdrawal during treatment and demographic variables, the null hypothesis for all associations shall be assumed.

## Methods

### Procedure

The Anxiety Online (now called Mental Health Online) platform consists of 4 centers: psychoeducational, assessment, treatment, and training. The psychoeducational center is a website that provides psychoeducational information about prevalence, symptoms, and treatments of anxiety disorders as well as links to useful resources. The assessment center contains the electronic psychological assessment screening system (e-PASS). The treatment center provides and manages the 5 anxiety treatment programs. The training center provides the eTherapist training programs and a health care practitioner portal. The online psychological assessment and referral program, e-PASS, includes a variety of demographic and personal questions, including the Kessler-6 measure of psychological distress, as well as the online diagnostic program. Individuals can access the Anxiety Online service from anywhere in the world provided they have an Internet connection. People complete the e-PASS if they want a psychological assessment and/or if they are interested in online treatment. Based on an individual’s response to some of the e-PASS questions, a person may be given a primary diagnosis and/or multiple secondary diagnoses. Those adults (18 years old or older) diagnosed with panic disorder, social anxiety disorder, generalized anxiety disorder, posttraumatic stress disorder, or obsessive-compulsive disorder are offered a 12-week self-guided or therapist-assisted treatment program (the therapist-assisted program is only available to Australian residents). To accommodate a formal withdrawal during treatment, an opt-out button within each account is provided. When this button is pressed, participants are asked whether they would like to complete the exit survey (8 questions about why they are withdrawing). Following the 12-week treatment cycle, patients are asked to complete e-PASS again. The posttreatment questions are essentially the same as the pretreatment questions. Patients are also encouraged to complete e-PASS at yearly intervals for 5 years following treatment program cycle completion (see [14] for more details). Those who wanted to undertake the e-PASS were first required to register and consent to the Anxiety Online terms and conditions [32]. The procedures for collecting and reporting of the Anxiety Online data were approved by the Swinburne University Human Research Ethics Committee. From the time of its launch to the public in October 2009 until January 2012, the e-PASS program had been accessed by 10,745 people.

### Online Questions/Questionnaire: Self-Report

As shown in Multimedia Appendix 1, a total of 24 demographic and personal questions and 2 items that screen for suicide risk and psychosis made up the questionnaire that preceded the online diagnostic program. After completing the questionnaire, the person then completed the online diagnostic program, which consisted of many questions and measures, including the commonly used Kessler-6 [33] for clinical assessment of mental health.

The Kessler-6 consists of 6 items measured on a 5-point Likert scale, measuring nonspecific psychological distress over the past 30 days. Normative data indicate that 71.7% of the population report low distress scores of 6-11, 16.6% of the population report moderate distress scores of 12-15, 7.16% of the population report high distress scores of 16-19, whereas 2.5% of the population report very high distress scores of 20-30 [33,34].

### Participants

**Figure 2:**
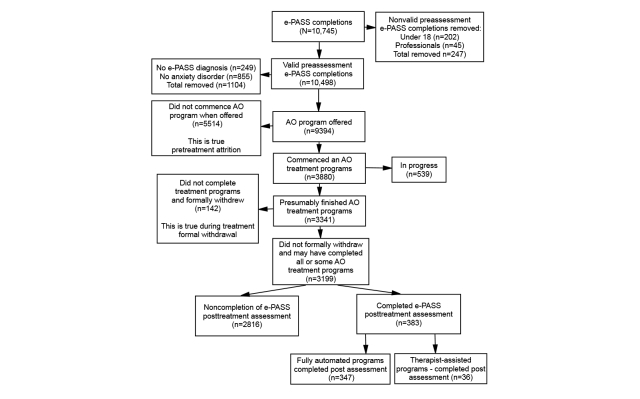
Recruitment and enrollment rate of participants to Anxiety Online (AO) website.

As shown in Figure 2, a total of 10,745 individuals completed the pretreatment assessment phase between October 2009 and January 2012. Some of those individuals were younger than 18 years (n=202) and some were professionals (n=45) who were exploring the assessment instrument. These 247 individuals were removed from the data leaving 10,498 valid completers of the e-PASS program. In addition, 249 individuals did not receive an e-PASS diagnosis and another 855 individuals who did not receive a diagnosis of any anxiety disorders were also removed from the dataset. This left 4771 (50.79%) with a primary diagnosis and 4623 (49.21%) with a secondary diagnosis of at least one of the anxiety disorders, for a total of 9394 e-PASS completers. All 9394 were offered a treatment program, although it was recommended that those with a primary diagnosis other than anxiety should seek help elsewhere. A total of 3880 (41.30%) individuals accepted and commenced a 12-week online treatment cycle, including 105 patients who selected the therapist-assisted path; 5514 individuals did not accept the offer of an anxiety treatment program. Of those who commenced treatment, 2321 (59.82%) had one of the anxiety disorders as their primary diagnosis, whereas 1559 (40.18%) had at least one anxiety disorder as their secondary diagnosis. Of those who did not commence treatment, 2450 (44.43%) had one of the anxiety disorders as their primary diagnosis, whereas 3064 (55.57%) had at least one anxiety disorder as their secondary diagnosis. The first part of the analysis in this study investigates the differences between the treatment commencers and those who choose not to start 1 of the 5 anxiety disorder treatment programs in terms of the pretreatment assessment variables described in Multimedia Appendix 1.

We considered treatment nonacceptance as pretreatment attrition in this study. At the time of analysis, there were 539 individuals still undergoing treatment; therefore, 3341 individuals either had or had not formally withdrawn from the 12-week treatment cycle. A total of 3199 individuals did not formally withdraw from their treatment program cycle, whereas 142 individuals formally withdrew during their treatment program cycle. Those who did not formally withdraw from the treatment cycle consisted of 1005 (31.42%) males aged between 18 and 78 years with a mean age of 38.43 (SD 12.23) years, and 2194 (68.58%) females aged between 18 and 81 years with a mean age of 35.81 (SD 11.87) years. The second part of the analysis investigates the differences between those who did not formally withdraw during treatment in comparison to those who did formally withdraw on the basis of pretreatment assessment measures. We defined the formal withdrawal during treatment rate as the proportion of individuals who formally withdrew from their treatment cycle relative to the total number of individuals who commenced the treatment program cycle.

### Analysis

The same method of analysis was used for analyzing pretreatment attrition and formal withdrawal during treatment. The initial univariate analyses used chi-square tests of association to determine which of the previously described variables have a significant relationship with attrition. A multivariate analysis was used to confirm the univariate results. Multivariate binary logistic regression analyses were performed with a forward selection approach used to identify the most important predictor variables. The final model, using only the most important predictor variables, was evaluated using a Hosmer-Lemeshow test. All analyses were conducted using SPSS version 20 (IBM Corp, Armonk, NY, USA).

## Results

### Pretreatment Attrition (Profile of Treatment Commencers)

In this study, there were 9394 individuals with a valid pretreatment e-PASS assessment who were offered a 12-week Anxiety Online treatment program. Only 3880 of these individuals actually commenced a treatment cycle, whereas 5514 individuals chose not to participate in any of the 5 Anxiety Online treatment programs, yielding a pretreatment attrition rate of 58.70% and a commencement rate of 41.30%.

As shown in Table 1, the chi-square tests of association showed 22 e-PASS pretreatment assessment variables were significantly associated with program commencement. Those who accepted and commenced treatment tended to differ from those who did not accept to enroll in the treatment program in several ways. On average, it was more likely that the treatment commencers had heard about the program through traditional media rather than the Internet; were seeking online assistance with the primary goal of finding a self-help program; were willing to provide consumer feedback; were married; were living in metropolitan areas; were employed full-time; had completed grade 12 schooling; were postgraduates; were nonsmokers; thought that they had adequate social support; rated their self-confidence as good; rated their quality of life as good; were prepared to take action or were in the process of making changes to deal with their mental health issues; said that they learn by reading; had lower pretreatment Kessler-6 scores; were slightly older; and had expressed concerns about their anxiety symptoms, but not about depression, eating disorders, or alcohol and substance abuse. This last characteristic is to be expected because individuals with a primary diagnosis other than anxiety were advised to seek treatment elsewhere.

**Table 1:** Predictor analysis for attrition categories for those who commenced treatment vs those who did not commence treatment (N=9394).

Variables		Attrition categories		Test of association		
		Did not commence treatment (n=5514)	Commenced treatment (n=3880)	χ^2^ (*df*)	*F*_1,9392_	*P*
**How did you hear about us?, n (%)**				134.4 (4)		.001
	Internet	2712 (49.18)	1694 (43.66)			
	Health professional	781 (14.16)	573 (14.77)			
	Friend/family	530 (9.61)	272 (7.01)			
	Media	768 (13.93)	878 (22.63)			
	Other	723 (13.11)	463 (11.93)			
**Reason for seeking online assistance, n (%)**				657.8 (1)		.001
	To complete one of the self-help programs	1694 (30.72)	2220 (57.22)			
Join research registry (yes), n (%)		1686 (30.58)	1270 (32.73)	4.1 (1)		.04
Provide consumer feedback (yes), n (%)		2444 (44.32)	1932 (49.79)	22.8 (1)		.001
**Gender, n (%)**				13.8 (1)		.001
	Male	1555 (28.20)	1232 (31.75)			
	Female	3959 (71.80)	2648 (68.25)			
**Relationship status, n (%)**				62.6 (6)		.001
	Married	1599 (29.00)	1389 (35.80)			
	Single	1693 (30.70)	1114 (28.71)			
	Cohabiting	1073 (19.46)	716 (18.45)			
	Not living together	669 (12.13)	375 (9.66)			
	Separated/divorced	351 (6.37)	217 (5.59)			
	Widowed	21 (0.38)	23 (0.59)			
	Other	108 (1.96)	46 (1.19)			
**Setting, n (%)**				28.3 (3)		.001
	Metropolitan	3335 (60.48)	2553 (65.80)			
	Regional	1485 (26.93)	895 (23.07)			
	Rural	630 (11.43)	398 (10.26)			
	Remote	64 (1.16)	34 (0.88)			
**Employment status, n (%)**				19.4 (6)		.004
	Employed full-time	2121 (38.47)	1611 (41.52)			
	Employed part-time	1359 (24.65)	930 (23.97)			
	Home duties	430 (7.80)	280 (7.22)			
	Disability support	223 (4.04)	142 (3.66)			
	Unemployed	664 (12.04)	396 (10.21)			
	Retired	106 (1.92)	101 (2.60)			
	Other	611 (11.08)	420 (10.82)			
**Highest level of schooling, n (%)**				32.8 (5)		.001
	None	66 (1.20)	37 (0.95)			
	Primary school	53 (0.96)	24 (0.62)			
	Secondary (grade 9)	157 (2.85)	98 (2.53)			
	Secondary (grade 10)	534 (9.68)	343 (8.84)			
	Secondary (grade 11)	490 (8.89)	243 (6.26)			
	Secondary (grade 12)	4214 (76.42)	3135 (80.80)			
**Postsecondary, n (%)**				144.0 (6)		.001
	None	855 (15.51)	492 (12.68)			
	Apprenticeship/trade	278 (5.04)	155 (3.99)			
	Certificate	754 (13.67)	387 (9.97)			
	Diploma	633 (11.48)	395 (10.18)			
	Undergraduate	869 (15.76)	492 (12.68)			
	Postgraduate	826 (14.98)	840 (21.65)			
	Other	1299 (23.56)	1119 (28.84)			
**Mental health concern, n (%)**				314.2 (6)		.001
	None	73 (1.32)	33 (0.85)			
	Anxiety	3445 (62.48)	3040 (78.35)			
	Stress	465 (8.43)	246 (6.34)			
	Depression	885 (16.05)	362 (9.33)			
	Substance abuse/alcohol	74 (1.34)	19 (0.49)			
	Eating	378 (6.86)	75 (1.93)			
	Other	194 (3.52)	105 (2.71)			
Currently receiving mental health assistance (yes), n (%)		2121 (38.47)	1415 (36.47)	3.9 (1)		.05
**Number of doctor visits, n (%)**				12.5 (2)		.002
	None	2310 (41.89)	1690 (43.56)			
	1-2 visits	2416 (43.82)	1732 (44.64)			
	3 or more	788 (14.29)	458 (11.80)			
Do you smoke? (yes), n (%)		1300 (23.58)	632 (16.29)	74.0 (1)		.001
Social support (yes), n (%)		2312 (41.93)	1775 (45.75)	13.5 (1)		.001
**Self-confidence, n (%)**				37.6 (4)		.001
	Very poor	511 (9.27)	264 (6.80)			
	Poor	1577 (28.60)	997 (25.70)			
	Neither	1990 (36.09)	1460 (37.63)			
	Good	1239 (22.47)	1008 (25.98)			
	Very good	197 (3.57)	151 (3.89)			
**Quality of life, n (%)**				28.7 (4)		.001
	Very poor	215 (3.90)	126 (3.25)			
	Poor	1019 (18.48)	592 (15.26)			
	Neither	1653 (29.98)	1123 (28.94)			
	Good	2213 (40.13)	1705 (43.94)			
	Very good	414 (7.51)	334 (8.61)			
**Making changes, n (%)**				84.9 (4)		.001
	Not interested	79 (1.43)	23 (0.59)			
	Neither	533 (9.67)	199 (5.13)			
	Prepared to take action	2808 (50.92)	2089 (53.84)			
	In process of making changes	1440 (26.12)	1118 (28.81)			
	Relapse	654 (11.86)	451 (11.62)			
**How do you best learn?, n (%)**				31.1 (3)		.001
	Hearing	361 (6.55)	222 (5.72)			
	Reading	1371 (24.86)	1164 (30.00)			
	Looking	1065 (19.31)	707 (18.22)			
	Doing	2717 (49.27)	1787 (46.06)			
Pre-Kessler-6 (total score), mean (SD)		18.35 (4.73)	16.89 (4.84)		212.86	.001
Age (years), mean (SD)		34.23 (11.90)	36.43 (12.07)		77.30	.001

As shown in Table 2, the final binary logistic regression with forward selection of predictors for pretreatment attrition contained 10 significant predictors. The Hosmer-Lemeshow goodness-of-fit test indicated an adequate model fit. When statistically controlling for the other variables in the model, we found significant odds ratios for the predictors reason, mental health concerns, postsecondary education, where first heard about Anxiety Online, pre-Kessler-6 score, stage of change, quality of life, relationship status, preferred method of learning, and smoking. We should note here that the same 10 predictors were also found to be significantly associated with pretreatment attrition using chi-square tests as shown in Table 1.

The odds ratios for enrolling in an Anxiety Online treatment program cycle in order of significance were: 2.9 times higher for individuals who gave “seeking to use one of the online self-help programs” as a reason for joining the program relative to all other reasons; 0.7 times as great for those who expressed concerns about eating and weight issues as for those who reported “none” for concerns; 1.29 times higher for those who completed undergraduate degrees and 0.81 times as great for those who hold other certificates as for those who reported having no postsecondary education; 1.35 times higher for those who heard about Anxiety Online from the traditional media relative to all other sources that are not listed in Table 3; 3% reduction in likelihood for each additional point an individual scored on the Kessler-6 total score; 2.16, 2.21, and 2.29 times higher for those who were prepared to make changes, reporting that they were already making changes, and who reported being in relapse and seeking further assistance, respectively, relative to those who were disinterested or indifferent; 1.92, 1.35, and 1.26 times higher for those who rated their quality of life as very poor, poor, and neither poor or good, respectively, relative to those who gave a rating of very good; 1.55 and 1.53 times higher for married and single individuals, respectively, relative to those reporting some other relationship status not listed in Table 3; 1.18 times higher for those indicating that they learn best by reading relative to those who said they learn best by doing; and finally, 1.19 times higher for those who identified themselves as nonsmokers relative to smokers.

**Table 2:** Binary logistic regression model for pretreatment attrition.

Variables		Wald	*df*	*P*	OR	95% CI
**Heard (reference group: other sources)**		34.59	4	.001		
	Surfing the net	0.07	1	.79	0.98	0.85-1.13
	Health professional	0.08	1	.78	1.03	0.86-1.22
	Friend or family	3.48	1	.06	0.83	0.67-1.01
	Media	12.65	1	.001	1.35	1.14-1.59
Reason (online self-help)(reference group: other reasons)		514.46	1	.001	2.90	2.65-3.18
**Relationship status (reference group: other relationship status)**		15.27	6	.02		
	Married	4.94	1	.03	1.55	1.05-2.27
	Single	4.65	1	.03	1.53	1.04-2.25
	Cohabiting	3.13	1	.08	1.42	0.96-2.10
	Not living together	1.07	1	.30	1.24	0.83-1.84
	Separated/divorce and not in relationship	1.52	1	.22	1.30	0.86-1.97
	Widowed and not in relationship	3.00	1	.08	1.91	0.92-3.95
**Postsecondary education (reference group: none)**		43.14	6	.001		
	Apprenticeship/trade certificate	2.28	1	.13	0.83	0.65-1.06
	Other certificates	5.45	1	.02	0.81	0.68-0.97
	Diploma	0.39	1	.53	0.94	0.79-1.13
	Current undergraduate	0.28	1	.60	0.96	0.80-1.13
	Completed undergraduate	9.47	1	.002	1.29	1.10-1.52
	Postgraduate degree	3.74	1	.05	1.16	1.00-1.348
**Mental health concerns (reference group: none)**		201.96	6	.001		
	Anxiety	1.92	1	.17	1.37	0.88-2.14
	Stress	0.44	1	.51	0.85	0.53-1.37
	Depression	1.15	1	.28	0.78	0.49-1.23
	Substance/alcohol abuse	3.68	1	.06	0.51	0.26-1.02
	Eating/weight issues	21.30	1	.001	0.30	0.18-0.50
	Other	0.11	1	.75	1.09	0.65-1.82
Smoke (nonsmoking) (reference group: smoking)		8.47	1	.004	1.19	1.06-1.34
**Quality of life (reference group: very good)**		21.72	4	.001		
	Very poor	16.64	1	.001	1.92	1.40-2.63
	Poor	7.47	1	.006	1.35	1.09-1.67
	Neither poor or good	5.83	1	.02	1.26	1.05-1.52
	Good	1.20	1	.27	1.10	0.93-1.31
**Making changes (reference group: disinterested or indifferent)**		22.89	4	.001		
	Neither here nor there	2.66	1	.10	1.55	0.92-2.62
	Prepared to take action	9.02	1	.003	2.16	1.31-3.58
	Already in the process of making changes	9.36	1	.002	2.21	1.33-3.67
	Relapsed and looking for additional assistance	9.79	1	.002	2.29	1.36-3.85
**Learning (reference group: doing)**		10.35	3	.02		
	By hearing	0.12	1	.73	0.97	0.80-1.17
	By reading	9.36	1	.002	1.18	1.06-1.34
	By looking/watching	0.87	1	.35	1.06	0.94-1.20
Pre-Kessler-6		30.01	1	.001	0.97	0.95-0.98
Constant		10.35	1	.001	0.29	

**Table 3:** Predictor analysis for categories for formal treatment cycle withdrawal.

Variables		Categories		Test of association		
		Not formally withdrawn from treatment (n=3199)	Formal treatment dropouts (n=142)	χ^2^ (*df*)	*F*_1,3339_	*P*
**How did you hear about us?, n (%)**				9.4 (4)		.05
	Internet	1344 (42.01)	71 (50.0)			
	Heath professional	475 (14.85)	19 (13.4)			
	Friend/family	227 (7.10)	15 (10.6)			
	Media	761 (23.79)	28 (19.7)			
	Other	392 (12.25)	9 (6.3)			
Reason for seeking online assistance				1.8 (1)		.19
Join research registry				0.0 (1)		.91
Provide consumer feedback				0.9 (1)		.63
Gender				0.1 (1)		.78
Relationship status				0.7 (3)		.87
Setting				5.1 (2)		.08
Employment status				4.3 (3)		.23
Highest level of schooling				0.5 (3)		.91
Postsecondary				2.2 (6)		.90
**Mental health concern, n (%)**				11.7 (3)		.009
	Anxiety	2510 (78.46)	103 (72.5)			
	Stress	204 (6.38)	8 (5.6)			
	Depression	302 (9.44)	13 (9.2)			
	Other	183 (5.72)	18 (12.7)			
Currently receiving mental health assistance				0.1 (1)		.82
Have you accessed mental health in last 12 months				0.3 (1)		.61
Have you ever accessed mental health				0.0 (1)		.85
Any diagnosed physical health condition				0.1 (1)		.80
Doctor visit				0.2 (2)		.89
Do you smoke?				0.1 (1)		.79
Do you drink alcohol?				2.2 (1)		.14
Adequate social support (yes), n (%)		1482 (46.33)	54 (38.0)	3.8 (1)		.05
Self-confidence				2.2 (4)		.71
Quality of life				4.5 (3)		.22
**Making changes (change), n (%)**				8.9 (3)		.03
	Not matter	171 (5.35)	15 (10.6)			
	Prepared	1722 (53.83)	73 (51.4)			
	Already	936 (29.26)	34 (23.9)			
	relapse	370 (11.57)	20 (14.1)			
How do you best learn?				1.3 (3)		.73
Age in years					0.04	.84
Kessler-6 (total scores)					0.01	.92

### Formal Withdrawal During Treatment (Predictors of Formal Dropouts)

There were 3880 individuals who commenced a 12-week Anxiety Online treatment program cycle. The number of individuals still undergoing treatment at the time of this analysis was 539; therefore, 3341 individuals were included in the analysis. A total of 142 individuals formally withdrew from their Anxiety Online treatment program cycle; therefore, 3199 individuals did not formally withdraw and potentially completed some or all of a 12-week treatment program cycle. The formal withdrawal during treatment rate was defined as the number of individuals who formally withdrew from their treatment program cycle in relation to the total number of individuals who commenced treatment. The formal withdrawal during treatment rate was 4.25% (142/3341) with 95.75% (3199/3341) not formally withdrawing during treatment. It is important to note that this rate is an underestimate of the true attrition because it relies exclusively on those who formally withdrew from the treatment program.

As shown in Table 3, the chi-square tests of association showed 2 pretreatment variables, mental health concerns and stages of change, were significantly associated with those who formally dropped out of treatment. Those who formally withdrew from the Anxiety Online treatment program cycle were, on average, less likely to express concern about anxiety and were less prepared to make changes in their lives to deal with their conditions than those who did not formally withdraw from treatment.

To simplify the interpretation of the odds ratios, we have reported the odds for not formally withdrawing from the treatment program. As shown in Table 4, the final binary logistic regression with forward selection of predictors for not formally dropping out of the treatment program contained 4 significant predictors. When statistically controlling for the other variables in the model, we found significant odds ratios for the predictors mental health concerns, adequate social support, quality of life, and stages of change. The Hosmer-Lemeshow goodness-of-fit test indicated an adequate model fit.

The odds of not formally dropping out of the Anxiety Online treatment program cycle in order of significance were: 2.34, 2.59, and 2.30 times higher for those who expressed concerns over anxiety, stress, and depression, respectively, relative to those who expressed concerns over other mental health issues; 2.62, 2.13, and 1.87 times higher for those who rated their quality of life as very poor/poor, neither poor or good, and good relative to those who gave a rating of very good; 1.70 times higher for those who reported having adequate level of social support; and 1.96 and 2.32 times for those who were prepared to make changes and those reporting that they were already making changes, respectively, relative to those who were disinterested or indifferent. Conversely, these odds ratios suggest that in general the likelihood of formally withdrawing from the treatment programs decreased for those who expressed concerns over anxiety, stress, and depression; viewed their quality of life as very poor/poor, neither good or poor, and good; reported adequate level of social support; and were already making changes or prepared to make changes.

**Table 4:** Binary logistic regression model for formal treatment withdrawal.

Variables		Wald	*df*	*P*	OR	95% CI
**Mental health concern (reference group: other)**		10.28	3	.02		
	Anxiety	9.83	1	.002	2.34	1.38-3.98
	Stress	4.65	1	.03	2.59	1.09-6.13
	Depression	4.75	1	.03	2.30	1.09-4.86
Social support (adequate)(reference group: not adequate)		7.38	1	.007	1.70	1.16-2.49
**Quality of life (reference group: very good)**		8.38	3	.04		
	Very poor and poor	7.74	1	.005	2.62	1.33-5.15
	Neither poor or good	6.02	1	.01	2.13	1.16-3.89
	Good	4.98	1	.03	1.87	1.08-3.24
**Making changes (reference group: disinterested or indifferent)**		7.66	3	.05		
	Prepared to take action	5.07	1	.02	1.96	1.09-3.53
	Already making changes	6.70	1	.01	2.32	1.23-4.38
	Relapsed and looking for additional assistance	1.33	1	.25	1.51	0.75-3.05
Constant		3.58	1	.06	2.28	

## Discussion

In this study, pretreatment attrition was defined as not accepting 1 of 5 Anxiety Online treatment programs, whereas formal withdrawal during treatment was defined simply as those who formally withdrew from their 12-week Anxiety Online treatment program cycle. The purpose of this study was to identify predictors of pretreatment attrition and predictors of those who formally withdrew or, conversely, those who did not formally withdraw from the treatment program cycle.

The results showed that the Anxiety Online program was found to have a pretreatment attrition rate of 58.7%. There are few studies that have reported pretreatment attrition for online or face-to-face treatment. Only 1 study reported a higher pretreatment attrition rate of 85% for individuals with social anxiety [31], whereas 2 studies reported approximately half the pretreatment attrition value of this study: Richards and Borglin [30] for a study on anxiety and depression reported a pretreatment attrition of 27% and Issakidis and Andrews [23] for a study on anxiety disorders reported a pretreatment attrition of 30.4%. Our higher pretreatment attrition may be because of the fact that Anxiety Online is an e-mental health service, whereas the other 2 studies were face-to-face clinic-based services. This is not surprising because public e-mental health treatment services tend to have higher treatment attrition rates [16-18].

The Anxiety Online treatment programs were found to have a during-treatment formal withdrawal rate of 4.3% (defined by those participants who formally dropped out of a program after commencement). This low rate could be related to our too-inclusive approach. The fact that we used those who did not formally withdraw during the 12-week treatment cycle may account for this low attrition rate. We do not have the data to show whether or not those who did not formally withdraw accessed all 12 modules, but we do know that they did not formally withdraw during the 12-week treatment cycle. It is possible that all patients who did not formally withdraw completed all 12 treatment modules, but it is equally possible that they did not work through all the modules. Therefore, further studies on the Anxiety Online data are required when module completion data becomes available. It should be emphasized that the diversity of attrition definitions in general and the approach we used in this work make any comparison of attrition rates problematic.

In this study, 24 demographic variables and one clinical measure of psychological distress were used to predict pretreatment attrition variables and formal withdrawal from treatment variables. Chi-square tests of association and binary logistic regression were used to relate these variables to pretreatment and during-treatment formal withdrawal. Results showed that the likelihood of enrolling in one of the Anxiety Online treatment programs increased for those who were seeking to use one of the self-help treatment programs; had an undergraduate degree; heard about the program via traditional media sources; were prepared to make changes, already making changes, or in relapse and looking for additional assistance; rated their quality of life as very poor, poor, or neutral rather than very good; married or single; reported learning best by reading; and those who identified themselves as nonsmokers. In general, these results should be expected. People who are seeking online self-help are likely to accept online treatment. If we extend the recent findings by Dahlstrom [35] and Edwards [36] that most undergraduates are comfortable with various communication technologies and favor the use of virtual communication in their education, then it is not surprising that the probability of enrolling in the online treatment program was greater for those who held undergraduate degrees; those who hold undergraduate degrees are likely to be more familiar and comfortable with the technology. It is not surprising that individuals who are prepared to or are making changes would enroll in treatment programs. The less people thought that their quality of life was good, the more likely they were to accept treatment. People who think they have a very good quality of life, regardless of mental health concerns, perhaps do not believe that treatment would further improve their lives. Because the Anxiety Online service is online and largely text-based, it is logical to find that the probability of enrolling in a treatment program increases for those who learn best by reading.

The odds of enrolling in the treatment program decreased for those who expressed concerns over eating and weight issues, probably because individuals with these problems presenting as their primary disorder were advised to find help elsewhere. Finally, those who reported greater psychological distress as measured by the Kessler-6 were less likely to accept the offer to enroll in the treatment program, probably because patients with higher scores may have felt too overwhelmed to commence treatment at this stage and may have opted for face-to-face treatment instead. Finally, it should be noted that some people who completed the e-PASS measures were not interested or ready to start treatment; rather, they just wanted an assessment.

On the other hand, results showed that the odds of formally withdrawing from treatment increased for those who did not express concerns about anxiety, stress, and depression; decreased for those who rated their quality of life as less than very good; decreased for those who reported having an adequate level of social support; and decreased for those who were prepared to make changes or were already making changes to improve their mental health. Participants who commenced the online anxiety treatment cycle would have received one of the anxiety disorder diagnoses as either a primary or secondary diagnosis. As such, it makes sense that those who expressed concerns about symptoms (anxiety, stress, and depression) that were congruent with their diagnoses would more likely not formally withdraw from the treatment cycle than those who expressed concerns about other issues that were not congruent with their diagnoses. That is, those who were less concerned about symptoms of anxiety, stress, and depression were more likely to formally drop out. Individuals who reported adequate social support were more likely not to formally withdraw from the program, perhaps because their social support resources provided a good safety net during times of stress. Moreover, such people may be more likely to have a positive outlook on life and perhaps greater potential for change.

As was the case in the pretreatment attrition predictors, individuals who perceived their quality of life as less than very good and who were willing or in the process of making changes were more likely not to formally withdraw from their treatment cycle. Individuals who reported a very good quality of life were more likely to formally withdraw from treatment. This is probably because participants who perceived their quality of life as very good were unlikely to think that treatment could further improve the quality of their lives. Finally, part of any treatment program is to make changes in one’s life; therefore, it is understandable why those who were not in the process of making or were unprepared to make changes were more likely to formally withdraw from their treatment program cycle.

Comparison of attrition rates between studies is problematic for 3 reasons. Firstly, definitions of attrition vary between studies. For example, the definition of treatment attrition in the literature varies from dropping out after one treatment session to dropping out after several treatment sessions or, as in this study, to those who formally withdraw during treatment. Although it seems logical to define attrition as simply noncompleters of a treatment program regardless of the number of treatment modules (or sessions in some studies) attended, it is often the case that only a few participants complete all treatment modules or drop out without formally withdrawing from treatment and, therefore, it becomes desirable to include individuals who attend at least most modules in the completers group. Moreover, some people may not need to complete all the modules to acquire what they need to improve [37,38]. Secondly, the studies being compared may involve very different treatments with very different durations of intervention. For these reasons, comparisons with existing studies should be approached with caution. The third problem with comparisons of attrition studies relates to the paucity of such studies that explain how attrition relates to demographic and treatment factors.

Comparing the predictor variables found in this study with previous findings is difficult because of the lack of studies on predictors of pretreatment and formal withdrawal, during treatment especially for online therapy, and the lack of consensus regarding predictors of attrition. However, in general our results are in agreement with Issakidid and Andrews [23] in finding significant relationships between pretreatment attrition and severity of symptoms. In relation to formal treatment withdrawal, sex, age, income, educational level, and comorbidity were not found to be significant predictors of formal withdrawal during treatment, which is contrary to the findings of several studies [22-26]. But these studies were conducted on clinical samples receiving clinic-based face-to-face treatment rather than e-mental health treatments and did not use our formal withdrawal from treatment approach. Also, perhaps our group of participants is more homogeneous in that the group is already biased by virtue of selecting to engage in online therapy and the group is likely to share the cluster of anxiety disorders. That is, a group of this kind has more characteristics in common. Consequently, formally dropping out of the treatment program cycle by such a group may depend largely on only transient and controllable attributes such as concern, or lack of concern, over anxiety and depression, perception of quality of life, and readiness, or lack of, to make changes, and characteristics of the surroundings such as the availability of adequate social support, rather than more permanent and less controllable attributes.

Future research should examine the difference between the characteristics of those who select online therapy and those who prefer the more traditional method of face-to-face treatment. It is likely that as a group, those who are more comfortable with the technology and seek to do things online differ from those who are less inclined to embrace technology on many attributes. Future research should also examine the demographic profiles of these 2 groups.

The lack of research and the inconsistent results on attrition predictor variables is primarily because of the recency of online therapy, the diversity of definitions of attrition, and the inclusion or exclusion of a large number of potential predictor variables. In the future and with the increase in e-mental health treatment, more research on attrition and predictors of attrition specific to online therapy is required.

## Multimedia Appendix 1

Self-report online questionnaire.

## Abbreviations

CBT: cognitive behavioral therapy
DSM-IV-TR: Diagnostic and Statistical Manual of Mental Disorders, 4th edition, text revision
e-PASS: electronic psychological assessment screening system
